# Circulating Irisin in Healthy Adults: Changes after Acute Exercise, Correlation with Body Composition, and Energy Expenditure Parameters in Cross-Sectional Study

**DOI:** 10.3390/medicina56060274

**Published:** 2020-06-04

**Authors:** Rudite Lagzdina, Maija Rumaka, Gita Gersone, Peteris Tretjakovs

**Affiliations:** Faculty of Medicine, Department of Human Physiology and Biochemistry, Riga Stradins University, LV-1007 Riga, Latvia; Maija.Rumaka@rsu.lv (M.R.); Gita.Gersone@rsu.lv (G.G.); Peteris.Tretjakovs@rsu.lv (P.T.)

**Keywords:** irisin, healthy adults, physical exercise, resting metabolic rate, energy expenditure, body composition

## Abstract

*Background and Objectives*: Skeletal muscles are considered to be the main source of circulating irisin, both at rest and during physical activity. The aim of this study was to investigate the connection between irisin, body composition, and energy metabolism in humans. *Materials and Methods*: Serum irisin concentrations before and after acute aerobic exercise on a treadmill in 84 healthy adults were measured and their association with body composition and energy expenditure (EE) (obtained from indirect calorimetry) was determined. *Results*: The total pre-exercise irisin concentrations in males and females were similar, but higher in females when expressed per body mass kg (*p* < 0.001). There was an association between pre-exercise irisin per body mass kg, visceral fat rating (rho = −0.52, *p* = 0.001), and lean tissue % (rho = 0.41, *p* < 0.05) in males and lean body mass index (LBMI) (rho = −0.59, *p* < 0.001) in females. The pre-exercise irisin concentration correlated with the resting metabolic rate (RMR) in both sexes (rho = 0.44 in males, rho = 0.36 in females; *p* < 0.05), but with walking, running, and the EE difference from RMR in running (Δ running EE) in males only (rho = 0.32 to 0.37, *p* < 0.05). There was no significant change in irisin concentration after exercise in 58% of participants, while it decreased in 23%, and increased in 19%. In male subjects with no change in irisin concentration after exercise, running (*p* < 0.05) and Δ running EE per body mass kg (*p* < 0.05) were higher than in those with decreased irisin concentration. *Conclusions*: These findings indicate that the association of irisin concentration with body composition and EE parameters has sex-dependent differences, and acute exercise can lead to various changes in post-exercise irisin levels.

## 1. Introduction

Irisin is a 112 amino acid peptide produced from its precursor transmembrane protein, fibronectin type III domain containing protein 5 (FNDC5), following the action of proteases. Irisin is a peroxisome proliferator-activated receptor (PPAR) γ coactivator 1α (PGC1α) dependent myokine that was initially discovered by Boström and colleagues in 2012 [1].

The majority of studies relate the function of irisin to subcutaneous fat browning and increased uncoupling protein 1 expression in adipose tissue, as well as to expression of glucose transporter-4 in muscles due to exercise and shivering thermogenesis [2,3]. The role of irisin in skeletal muscle metabolism is thought to be related to regulation of the genes associated with the determination of the energetic reactions of cells [4]. Recently, an irisin receptor was identified through which bone remodeling and thermogenesis effects are mediated, clarifying the underlying mechanisms of irisin [5]. Studies in human and animal models describe irisin as a potential biomarker, as well as a protective and therapeutic agent in case of cardiac muscle, liver, brain, pancreas, bone, and ovary diseases [6,7,8,9,10,11].

In skeletal muscles, among the genes of which expression can be stimulated by physical exercise is the FNDC5 gene, and the exercise effect on the irisin level has been investigated in several studies. One study, conducted with the aim to validate whether the data on irisin function in rodents could be extrapolated to humans [12] failed to detect FNDC5 gene activation in human muscle biopsies after 10–11 weeks training and observed no effect of irisin or its precursor, recombinant FNDC5, on the brightening of primary human adipocytes. However, it is still thought that the secretion of irisin is influenced by physical activity (PA) and many exercise studies examining irisin are ongoing.

Studies on irisin level changes after long-term training reported various results, such as increase in circulating irisin after 10 and 12 weeks of aerobic training [1,13], no significant changes [14,15,16], or a decrease in irisin concentration [17].

Current data on the impact of acute exercise on irisin secretion are inconsistent. A meta-analysis of 10 purposely selected studies concluded that the irisin level increases by approximately 15% in response to acute resistance or aerobic exercise lasting at least 10 min [18], while in a study performed in Finland in adult men, there was no significant difference in irisin levels after one hour low intensity aerobic or heavy intensity exercise session [19]. In another study, after the strength training no immediate changes were observed, but after the endurance session the irisin level increased immediately [20]. Tsuchiya and colleagues demonstrated that after a single bout of exercise there was a decrease of irisin level immediately after the low intensity exercise, but no significant change as a result of the high intensity exercise [21]. In a study by Daskalopoulou et al. the irisin level changed following two different intensity exercises, with the highest increase after the maximal workload on a cycle ergometer [22]. Furthermore, studies found an increase in the irisin level immediately after short bout of cycling and running in healthy adults [14,23], but in the sample collected 10 min after exhausting cycling irisin level returned to baseline [23].

Although the highest concentration of irisin is found in the skeletal muscles [15], it can be produced in a variety of tissues. Published data about the association of circulating irisin level with body composition parameters in adults are inconsistent. Some studies have demonstrated higher irisin levels in individuals with higher body weight, BMI, and fat mass [24,25,26], and in subjects with a normal BMI but increased fat percent [27]. Moreno-Navarrete et al. found lower levels of irisin in overweight and obese individuals [28]. The concentration of irisin was also positively associated with bicep circumference, fat free mass [15], and lean body mass for both sexes [14].

In tissue metabolism, irisin stimulates metabolic activity of fat tissue and increases uncoupling protein 1 (UCP1) in the mitochondrial membrane; this functions to permit proton diffusion back to the mitochondrial matrix without concomitant ATP synthesis, which in turn stimulates cellular respiration and expands more energy to pump protons and restore the proton gradient across the inner mitochondrial membrane. Although this process increases oxygen consumption, the energy that is obtained mainly dissipates as heat [1]. Irisin also increases the basal metabolic activity of skeletal muscle cells in vitro following 24 h of irisin treatment, as well as increasing mitochondrial oxygen consumption during chemically induced peak oxidative metabolism [3]. Moreover, irisin also stimulates expression of genes that mediate an increase in fat and muscle metabolism [1,3]. A positive relationship of irisin with calculated RMR in females with different BMIs was found in the work of Pardo et al. [26], and the association of RMR with irisin concentration in blood is not well characterized in males. Swick used calorimetrically determined daily energy expenditure for postmenopausal women and found that the irisin level correlation with energy expenditure was only true for subjects with greater energy expenditure than those predicted from calculations using FFM [29].

## 2. Materials and Methods

The aim of this study was to add to currently limited knowledge about connection of irisin and energy metabolism in humans, to measure the circulating levels of irisin in the blood of healthy adults at rest and immediately after an acute aerobic exercise bout. The study involved the participants of both sexes in order to analyze the concentration of circulating irisin in relation to body fat, lean tissue, energy metabolism at rest and during exercise, and to compare the results in males and females.

### 2.1. Study Subjects

The study subjects were 86 adult volunteers of both sexes who met the following inclusion criteria: age 20–50 years, healthy (no acute or chronic medical conditions), and not using any medication during the study. The study was approved by the Ethics Committee of Riga Stradins University, and informed consent was provided by each participant upon study entry, No. 14/31.10.2013, 31 of October 2013.

### 2.2. Body Composition and Metabolism Measurements

To obtain more precise results, participants arrived in the laboratory in the morning after an overnight fast with limited PA before the test. All measures of the study protocol were performed on the same day between 8:00 a.m. and 10:00 a.m. First, the body height of each participant was measured to the nearest 1.0 cm and a Tanita MC-180 MA (Tanita Corp., Tokyo, Japan) multi-frequency bioimpedance analyzer was used to measure body mass and assess body composition, including fat and muscle mass segmental distribution in each extremity and trunk. The visceral fat level was determined and expressed as a rating. According to the assessment criteria defined by the device manufacturer, a rating 1 to 12 indicates a healthy level, while 13 and higher indicates an excess level of visceral fat. The BMI and lean body mass index (LBMI) were also calculated.

RMR determination and exercise tests were conducted, during which energy metabolism was monitored with indirect calorimetry, and respiratory gas analysis was performed on a breath-by-breath basis using a gas analysis system (Oxycon Pro, Carefusion, Germany). RMR measurements were performed in subjects remaining in the supine position, considering the guidelines for measurement of RMR in healthy adults [30]. From 30 min of data, the results of the initial 5 min were discarded, and the coefficient of variation (COV) for VO_2_ and VCO_2_ of the remaining period was calculated to determine if a steady state was achieved. The 5-minute period with the lowest (≤10%) COV was used to calculate the RMR.

Participants then performed 5 min of walking and running bouts on a treadmill with constant speeds of 4 and 8 km/h, respectively. The consecutive 60-second period with the lowest COV (≤10%) for VO_2_ and VCO_2_ during the last three minutes of each exercise was used to calculate the EE during walking and running. The EE difference from RMR (∆EE) for both exercises, and the relative ∆EE, when expressed on body mass kilogram, were calculated.

### 2.3. Irisin Measurement

Venous blood samples were obtained directly before and 5 min after the last bout of physical activity. After withdrawal, blood samples were centrifuged, serum collected, and stored at −80 °C until analysis. The irisin concentration was determined using a commercial enzyme-linked immunosorbent assay (ELISA) protocol (Irisin, Recombinant (Human, Mouse, Rat, Canine) (cat. no.: EK-067-29), range 0.1–1000 ng/mL, linear range 1.8–25.3 ng/mL, intra-assay variation: <10%, Phoenix Pharmaceuticals, Inc., Burlingame, CA, USA), rated as the best among currently available ELISA kits [31]. The same plate was used to analyze all irisin samples from each participant.

### 2.4. Statistical Analysis

In order to detect the presence of the outliers the interquartile range rule was used and data from two female participants were excluded from the final analysis due to extreme outliers in resting energy expenditure values in one, and in irisin levels in the other.

Statistical analysis was performed using SPSS Statistics v. 22.0 (IBM Corporation, USA) with significance set at *p* < 0.05. The analysis included descriptive statistics for the entire sample, stratified by sex in all cases. The results are presented as means and standard deviations or medians with interquartile ranges. Parameter differences between sexes were assessed using an independent samples *t*-test in cases of Gaussian data distribution and the Mann–Whitney and Kruskal–Wallis tests in cases of non-Gaussian data distribution. Spearman’s test was used to assess the correlation between variables.

## 3. Results

The study sample that was valid for analysis consisted of 84 participants and contained more women (52%) than men. The mean age in both sexes was similar, but the height, body mass, and BMI were significantly higher in the male group (*p* < 0.05 by independent-samples *t*-test), as shown in Table 1.

The measured serum irisin levels were similar in males and females; however, the relative pre- and post-exercise concentrations of irisin per body mass kg were higher in females (Table 2). There were no statistically significant differences between the corresponding pre-exercise irisin and post-exercise irisin results, neither in males nor in females (paired samples *t*-test, *p* > 0.05).

Individual responses to exercise included no changes, or increased or decreased serum irisin concentrations. To quantify the measured changes, Δ irisin was calculated as (1)

Δ irisin = ([post-exercise irisin − pre-exercise irisin]/[pre-exercise irisin]) × 100%(1)

**Figure 1:**
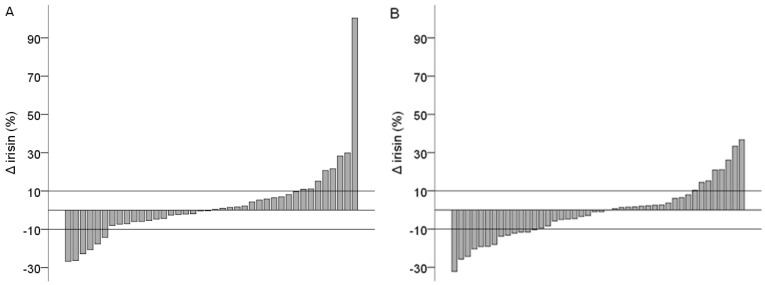
Change in blood irisin concentration after acute exercise in males (**A**) and females (**B**).

Depending on the Δ irisin result, the study sample was divided into three groups (Figure 1A,B), based on the cut-off value of the manufacturer’s defined precision range of intra-assay variation (<10%): Δ irisin group I, if the post-exercise irisin concentration decreased by more than 10% with respect to the pre-exercise value; Δ irisin group II, if changes were in the range plus to minus 10%; and Δ irisin group III, if the post-exercise irisin concentration increased by more than 10% with respect to the pre-exercise value. The Δ irisin group II included 58% (*n* = 49) of participants (65%:52%, M:F), Δ irisin group I included 23% (*n* = 19) (15%:30%, M:F), and Δ irisin group III included 19% (*n* = 16) of participants (20%:18%, M:F).

Initial correlation analysis revealed weak to moderate not statistically significant relationship between lean tissue, LBMI, visceral fat range, and total pre- and post-exercise irisin concentrations. Neither in males nor in females were clear relationships between BMI, body fat %, and irisin concentrations detected. In addition, no statistically significant differences in body composition parameters were found between the three Δ irisin groups (*p* > 0.05, Kruskal–Wallis test).

In order to test whether the relative pre-exercise irisin values per body mass kg were related to body composition, Spearman’s correlation test was performed (Table 3). Only in females was a negative association observed between LBMI and irisin. Furthermore, the percent of body lean tissue correlated positively, while visceral fat rating correlated negatively with irisin concentration in both males and females; however, only the male results were statistically significant. Association of BMI and body fat % with irisin concentration was not analyzed, since the body mass was a confounder in these relationships.

First, energy expenditure results were analyzed between sexes using the Mann–Whitney test. Compared to females, males demonstrated a higher RMR (1.4 ± 0.1 vs 1.0 ± 0.1 kcal/min, *p* < 0.0001), walking EE (4.6 ± 0.5 vs 3.8 ± 0.6 kcal/min, *p* < 0.0001), running EE (11.7 ± 1.3 vs 9.7 ± 1.3 kcal/min, *p* < 0.0001), Δ walking EE (3.3 ± 0.5 vs 2.7 ± 0.5 kcal/min), and Δ running EE (10.4 ± 1.3 vs 8.7 ± 1.2 kcal/min, *p* < 0.0001), Δ EE expenditures were calculated (2)

Δ walking EE = walking EE − RMR; Δ running EE = running EE − RMR(2)

When the absolute values were expressed per body mass kg the outcome was the opposite in that females used more energy in both walking and running exercise than males, and the difference was statistically significant for both running EE per body mass kg (*p* = 0.04) and Δ running EE per body mass kg (*p* = 0.02). Spearman’s correlation test detected that in both sexes, the RMR per kg strongly correlated with the percentage of lean tissue in the body (rho = 0.60 in males, rho = 0.57 in females; *p* < 0.000).

**Figure 2:**
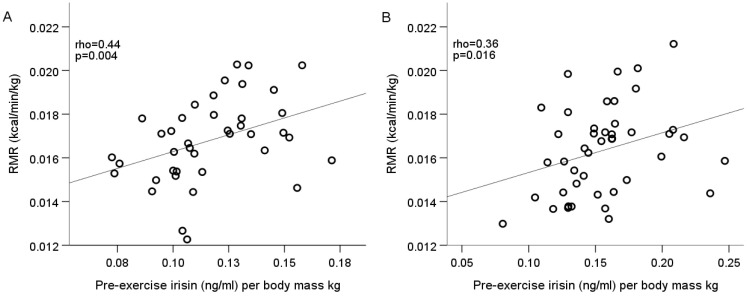
Pre-exercise irisin correlation with RMR in males (**A**) and females (**B**).

The relationship between irisin concentration and resting, walking, and running energy expenditure parameters in both sex groups was found to be positively correlated with pre-exercise irisin concentration per body mass kg and RMR value per body mass kg (Spearman’s rho = 0.44, *p* = 0.004 in males; rho = 0.36, *p* = 0.016 in females) (Figure 2A,B).

**Figure 3:**
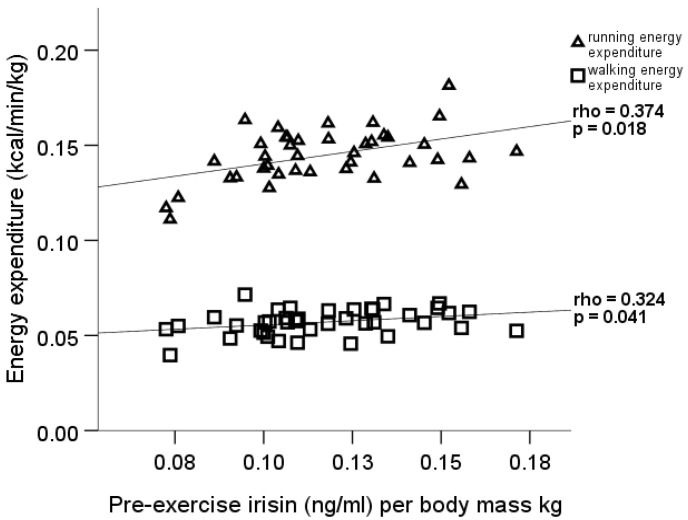
Pre-exercise irisin correlation with walking and running energy expenditure in males.

**Figure 4:**
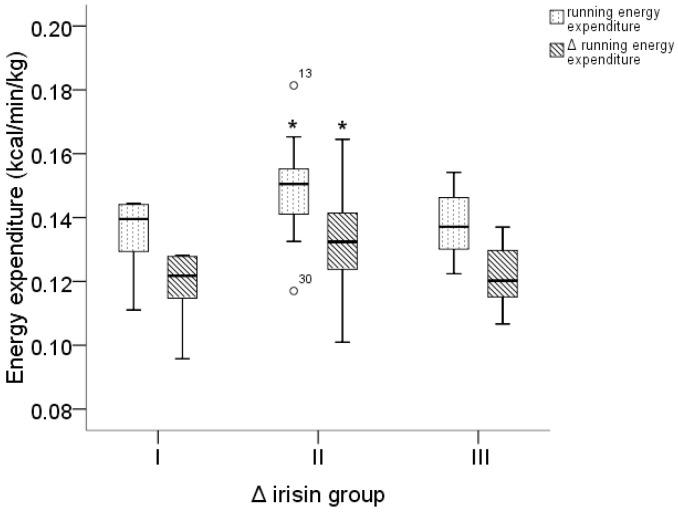
Distribution of running and Δ running energy expenditure in three Δ irisin groups in males. (* *group II* vs. *group I*).

Furthermore, the pre-exercise irisin concentration per body mass kg positively correlated with walking (rho = 0.32, *p* = 0.041), running (rho = 0.37, *p* = 0.018), and Δ running EE (rho = 0.35, *p* = 0.028) values per body mass kg in males only (Figure 3). The Kruskal–Wallis test and the following post-hoc analysis found statistically significant differences in males between the values of running EE per body mass kg and Δ running EE per body mass kg in the three Δ irisin groups. Results showed that the male participants in the Δ irisin group II had higher running EE per body mass kg (*p* = 0.042, 95% CI = −0.0004 to −0.0284) and Δ running EE per body mass kg (*p* = 0.047, 95% CI = −0.00015 to −0.02707) than the Δ irisin group I participants (Figure 4).

## 4. Discussion

The current study investigated the irisin concentration in healthy adults of both sexes. Since the pre-exercise irisin concentration was measured in blood samples obtained at the same time of day after the standardized preparation of participants, the results could be interpreted as a typical, basal irisin plasma concentration. Due to evidence that the irisin concentration may vary significantly depending on the utilized type of commercial kit, comparison of the results is possible only with studies that use the same type of kit. The average pre-exercise irisin level was consistent with the baseline level found in healthy individuals in the KarMeN-study [32], in a study of adipose women of a similar age reported by Winn et al. [33], a middle aged Chinese population [34], and even similar to the values measured in pregnant women between the second and third trimester of normal gestation [35]. However, the values that we report in the current study were almost 40 times lower than the baseline plasma irisin level determined in a small study sample of similar characteristics in Norway [20], and slightly lower than the baseline level detected in a group of adolescents [36]. In our study, there were no statistically significant differences between absolute male and female irisin serum concentrations, in neither the pre-exercise nor post-exercise blood sample. In the aforementioned studies (if measured), the baseline irisin did not differ between sexes. A higher value of irisin was observed in females in the present study when expressed per body mass kg and LBM, as was also observed in the study by Anastasilakis and colleagues [14] suggesting a possible influence of sex-specific factors on irisin secretion. 

In the current study, we found no significant relationship between total serum irisin concentration and the amount of irisin producing body tissue—lean or fat body mass, neither in common nor in sex-stratified correlation analysis. However, our results revealed that the specific interaction between the basal irisin level per body mass kg, and the amount of a particular body tissue type in healthy adults are directly correlated with lean body mass and inverse with fat tissue, notably with visceral fat. These associations are in line with the findings of a cross-sectional study in Korean adults, which confirmed the negative relationship between visceral fat and irisin, and that the higher skeletal muscle mass to visceral fat area ratio was associated with higher irisin levels [37]. In rat models, it has been demonstrated that adipose tissue, depending on the anatomical location, has distinct activity with respect to irisin secretion, while the subcutaneous fat cells secrete considerably more circulating irisin than visceral or fat cells in other locations [38]. To the best of our knowledge, there are no similar data regarding human fat cells. However, if we assume that the same principle applies to humans, then the lack of sex difference in absolute irisin concentration in the present study could be explained by the typically increased subcutaneous fat mass in women, which may have led to higher secretory activity, and resulted in higher irisin concentration expressed per body kg in females.

The current study demonstrated no association between total irisin concentration and BMI in both sexes. As BMI is widely accepted as an indicator of healthy body composition, which increases mainly due to body fat accumulation, the tendency could be in concordance with the statements that the primary source of circulating irisin is skeletal muscles, and to lesser extent, body fat tissue [38]. In obese individuals, subcutaneous and visceral adipose tissue show decreased FNDC5 gene expression and consequently lower concentrations of circulating irisin [28]. A number of previous studies, mainly involving healthy participants within a normal BMI range, reported no association between irisin and BMI [14,22]. Moreover, in a study with a control group, irisin positively correlated with BMI and body fat in both subgroups, in runners with normal BMI and in controls with increased BMI [39]. In particular study groups—especially in those with overweight and obese participants [31], and also across the groups with a wide BMI spectrum, including both anorexia nervosa and extremely adipose patients—irisin levels directly correlated with body mass, BMI, fat, and fat-free mass [25,26], indicating possible modification of irisin secretion from muscle and fat tissue when body metabolism and tissue proportions are changed. Besides the isolated effect on irisin secretion, alterations in production of many other myokines and adipokines, and their interaction on target cells have to be considered in each case [4].

A direct association between RMR and pre-exercise irisin values when expressed per body mass kg was the only significant relationship regarding energy metabolism that we observed in participants of both sexes. These data support positive correlation between lean body mass, RMR, and irisin secretion and can be attributed mainly to increased mitochondrial activity in skeletal muscles and stimulation of thermogenesis lipolysis, as well as increased energy expenditure in adipose tissue [40]. The lack of association between irisin and exercise energy expenditure parameters in females might indicate sex-related differences in the regulation of energetic metabolism. However, male subjects, whose pre-exercise irisin concentration was greater, metabolized energy to a greater extent in order to maintain walking and running activity. The most likely explanation would be irisin and energy expenditure correlation with individual body mass, but this was not relevant in our results since values per body mass kg were analyzed. In addition, other potential reasons—such as physical fitness status and habitual PA—were not confirmed as determinants of basal irisin levels in various studies [14,32]. Moreover, in men, an inverse relationship between basal irisin and exercise capacity assessed by peakVO_2_ was detected in large study involving healthy volunteers [41].

Despite the expected increase in irisin after exercise, our results for various Δ irisin are in line with the inconsistent findings of other studies that reported either no or various irisin concentration changes immediately after exercise intervention [16,19]. Further detailed analysis of current Δ irisin data allowed us to conclude that in men there is a certain relationship between the direction of irisin changes and energy expenditure. Respectively, male subjects without relevant differences between pre- and post-exercise irisin levels (Δ irisin group II) used more kcal in running per body mass kg, and their energy expenditure during running increased significantly more with respect to RMR than in those whose irisin levels decreased after exercise bouts. This could be explained by increased expression of various genes that are involved in cell energetics and mitochondrial biogenesis observed in in vitro skeletal muscle cells incubated with irisin [3]. However, this study reported the results in animal cell models, and the mechanism of irisin action in energetic metabolism in human real-body conditions could be more complex and represents a challenge for further research.

### Study Strengths and Limitations

One of the strengths of our study is that the selection of participants of both sexes allowed us to compare responses in both males and females. Furthermore, since all participants were apparently healthy, their body composition parameters were distributed around the range of normal values and testing of all the subjects occurred following an identical protocol and at the same time of day. This allowed us to interpret the pre-exercise irisin results as typical basal irisin concentrations for healthy persons, and the secretion responses as typical to a given physical load. In our study, the resting metabolic rate and energy expenditure were measured using indirect calorimetry which allowed us to operate with the real data for our subjects. In addition, the irisin concentration was determined using a test kit that is widely considered to be the most accurate. The lack of control group could be considered as one of the main limitations of our study. The availability of irisin results from control subjects in corresponding time periods would prove whether the observed irisin concentration changes were only exercise-induced or if they simply represented a natural fluctuation of irisin levels. Furthermore, the method we used for irisin measurement in serum samples did not provide the option to differentiate tissue circulating irisin or its fractions. Skeletal muscles in general are considered as the main secretory tissues for irisin; however, other tissue types, particularly fat tissue, also produce irisin. This limits our ability to provide a precise explanation of the origin of the irisin in different male and female results relative to the muscle and fat mass. In addition, we were also unable to definitively confirm which tissue type (skeletal muscle, subcutaneous, visceral fat) was predominantly responsible for the observed correlations; assuming that each tissue type, in case their proportions are different, could alter its secretory activity and lead to the observed associations with pre-exercise irisin concentration in men. The final limiting factor is that we only used the post-exercise blood sample for irisin measurement, which poses a question about the right time interval to assess irisin changes in blood after exercise.

## 5. Conclusions

The results of the current study suggest that the association of irisin concentration with body fat and lean tissue amount has sex-dependent differences, and that acute exercise can lead to various changes in irisin level in healthy adults. In participants of both sexes, a higher pre-exercise irisin level was associated with a higher resting metabolic rate, while in males it was also associated with higher energy expenditure during exercise. However, in males, no change in irisin levels after exercise was related to higher running energy expenditure. Since the direction of this relationship is unclear, and the magnitude of the changes of circulating irisin concentration could be considered functionally significant, further studies are needed to determine the role and regulation of irisin in humans.

## Figures and Tables

**Table 1:** Anthropometric and body composition measurements of the study participants.

Variable (units)	Male Mean ± SD (min–max) *n* = 40	Female Mean ± SD (min–max) *n* = 44
Age (years)	31.2 ± 5.3 (21–46)	29.9 ± 6.4 (21–49)
Height (cm)	184 ± 7 (170–198)	169 ± 5 (158–188)
Body mass (kg)	81.5 ± 9.7 (63.7–99.4)	64.3 ± 7.6 (50.1–79.0)
BMI (kg/m^2^)	24.2 ± 2.5 (19.6–31.9)	22.3 ± 2.7 (17.6–29.7)
Total fat tissue (kg)	12.8 ± 5.2 (3.5–24.3)	15.6 ± 4.9 (8.5–27.9)
Body fat (% from body mass)	15.3 ± 5.1 (5.2–26.4)	23.9 ± 5.3 (15.0–35.7)
Visceral fat (rating) ^1^	3.0 (2.3–6.0)	2.0 (1.0–3.0)
Lean body mass (kg)	68.8 ± 6.7 (53.9–83.0)	48.7 ± 4.4 (39.9–60.9)
Total muscle tissue (kg)	65.3 ± 6.4 (51.2–79.0)	46.2 ± 4.2 (37.8–57.85)

^1^ Results presented as median with IQR.

**Table 2:** Blood irisin concentration.

Variable (units)	Male Mean ± SD *n* = 40	Female Mean ± SD *n* = 44	*p*-Value ^1^
Pre-exercise irisin (ng/mL)	9.5 ± 1.9	9.9 ± 1.9	0.400
Post-exercise irisin (ng/mL)	9.6 ± 2.1	9.7 ± 2.0	0.943
Pre-exercise irisin per body mass kg (ng/mL)	0.12 ± 0.02	0.16 ± 0.03	<0.001
Post-exercise irisin per body mass kg (ng/mL)	0.12 ± 0.03	0.15 ± 0.03	<0.001

^1^*p*-value shows differences between sexes (Mann–Whitney test).

**Table 3:** Bivariate correlation between pre-exercise irisin and body composition parameters.

Variable (units)	Male Pre-Exercise Irisin (ng/mL) Per Body Mass kg	Female Pre-Exercise Irisin (ng/mL) Per Body Mass kg
LBMI (kg/m^2^)	−0.15	−0.59 **
Lean tissue (% from body mass)	0.41 *	0.28
Visceral fat (rating)	−0.52 **	−0.26

* p < 0.05, ** p ≤ 0.001.
